# Gender Stereotypes among Teachers and Trainers Working with Adolescents

**DOI:** 10.3390/ijerph182412964

**Published:** 2021-12-08

**Authors:** Adrián Mateo-Orcajada, Lucía Abenza-Cano, Raquel Vaquero-Cristóbal, Sonia M. Martínez-Castro, Alejandro Leiva-Arcas, Ana María Gallardo-Guerrero, Antonio Sánchez-Pato

**Affiliations:** 1 Faculty of Sport, Catholic University San Antonio of Murcia (UCAM), 135, 30107 Murcia, Spain; amateo5@ucam.edu (A.M.-O.); aleiva@ucam.edu (A.L.-A.); amgallardo@ucam.edu (A.M.G.-G.); apato@ucam.edu (A.S.-P.); 2 Faculty of Social Sciences and Communication, Catholic University San Antonio of Murcia (UCAM), 135, 30107 Murcia, Spain; smmartinez@ucam.edu; 3 Olympic Games Center, Catholic University San Antonio of Murcia (UCAM), 135, 30107 Murcia, Spain

**Keywords:** age, coaches, professors, sport, stereotyped behavior, years of experience

## Abstract

Previous scientific literature has not determined the influence exerted by trainers and teachers of adolescents on the development of gender stereotypes in sport. For this reason, the aims of the present research were to establish differences in gender stereotypes in sport among teachers and trainers as a function of profession and sex and to analyze the influence of age and years of experience of male and female trainers and teachers on the gender stereotypes in sport. For this purpose, 127 teachers and trainers completed the questionnaire “gender beliefs and stereotypes towards physical activity and sport”. The results showed a significantly higher score of the teachers in “beliefs about physical activity and gender” (*p* = 0.048) and of the trainers in “physical education classes and gender” (*p* = 0.006). Concerning sex, women showed higher scores in “sport and gender” (*p* = 0.005), and men in “beliefs about physical activity and gender” (*p* = 0.045). Regarding covariates, age showed significant differences in “sport and gender” (*p* = 0.029), with female teachers showing higher values with respect to female trainers and male teachers, while years of experience showed differences in “beliefs about sport and gender” (*p* = 0.044), with male teachers showing higher values than male trainers and female teachers.

## 1. Introduction

For decades, the practice of sport has been characterized by the presence of gender stereotypes that have led to consider sport as a purely masculine field in which women were unimportant [1]. However, some sports practices have been classified as feminine for years because only women practiced them, clear examples being some individual sports or body expression and aesthetics such as rhythmic gymnastics or swimming [2,3,4]. Progressively, the role of men and women in sport has changed, so that they now occupy similar positions in sports organizations, although gender differences are still present [5,6], and their participation rates in certain sports have increased [7,8]. Even so, a large part of sports activities are still marked by gender, which leads men and women to adjust their participation in the most stereotyped sports, even unconsciously [9].

In adolescents, previous scientific research has shown similar results to those found in the adult population, suggesting that gender stereotypes present in some sports may influence who participates in a sport and how it is viewed by others [10]. This has led boys to participate in sports traditionally considered masculine, such as those characterized by strength and speed, and girls to participate in feminine sports, such as those characterized by flexibility and aesthetics [2,3]. Fear of being judged and the perception of differences in the possibilities of practice between boys and girls are some of the reasons that lead many adolescent girls not to practice physical activity, even though they enjoy it [11].

This situation is worrying, but the reasons why adolescents present gender stereotypes related to sports practice seems to have a multifactorial origin, with previous research suggesting family, educational, and sports environments as the main elements in the transmission of certain stereotypes [12,13,14]. In the educational field, numerous proposals have been presented out in recent years to try to eliminate gender stereotypes, but a sexist idea of gender continues to be transmitted to young people through linguistic and visual elements present in textbooks [15,16], as well as through posters located in compulsory secondary education centers, whose images continue to refer to classic gender stereotypes. In fact, male sports practice is given special prominence in the images included in the materials found in educational centers, leaving female sports practice in a less privileged place, regardless of whether the linguistic messages present in these posters promote equality [17]. Physical education classes are no exception, and gender segregation and gendered discourses continue to exist within them, teachers being aware that in their classes there are numerous gender stereotypes, transmitted by themselves through biased and sex-segregated language, but unable to modify their behaviors due to a lack of awareness and understanding of gender equity that will allow them to offer an inclusive learning experience to all students [13,18,19]. This situation leads to teachers not attributing to themselves the reproduction of these stereotypes and commonly giving responsibility to parents and peers [20].

With respect to the sports field, previous research has shown the presence of gender stereotypes in managers and technical staffs. The presence of these stereotypes is indicated by the lower presence of women in coaching staffs, with men holding the most important positions [21,22]. This is due to the fact that in recent years, the reconciliation of work and family life has been a challenge for many women, since in certain societies the stereotype that women must attend to family demands to a greater extent than men is still present [23,24,25], as well as to the lack of female mentors of the same sex that makes it difficult for women to take an interest in the job as coaches [26]. Previous studies have shown that the gender of the members of the coaching staff, and specifically of the coach, is relevant in the hidden curriculum transmitted to athletes [14,27], being composed of the values, norms, preferences, or beliefs that professionals working with adolescents transmit to them through daily routines and the interaction established between them [28]. However, no research has analyzed the perception of trainers’ gender stereotypes in sport, despite their great influence on the players. Not in vain, the punctual programs involving young people and their trainers to favor gender equity in sport seem to show very little relevant effects on the latter [29], demonstrating the endurance of the stereotypes transmitted in the hidden curriculum.

Given the importance of the role that teachers and coaches play with young athletes and the knowledge that is currently available about the gender stereotypes present in both groups, research is needed to analyze the specific areas and dimensions in which coaches and teachers present these gender stereotypes and to analyze whether this phenomenon is different depending on the sex, age, or years of experience of the teachers or coaches, in order to design training programs that address gender stereotypes in a more specific way according to the work environment. For this reason, the aims of the present research were to establish the differences in gender stereotypes among teachers and trainers according to profession and sex and to analyze the influence of age and years of experience on the gender stereotypes of trainers and teachers.

## 2. Materials and Methods

### 2.1. Design

The design of this study is descriptive and transversal. The design and development of the manuscript were carried out according to the STROBE statement [30]. Before the study began, approval was obtained from the institutional ethics committee (code CE071924). The teachers and trainers who participated in the research signed an informed consent form at the beginning of the study, where they were informed of the aims of the study, as well as of the process of data collection and the confidentiality of the data obtained.

### 2.2. Participants

The sample size was calculated using Rstudio 3.15.0 software (Rstudio Inc.: Boston, MA, USA). The significance value was set at α = 0.05. The standard deviation (SD) was established in relation to previous studies on gender stereotypes in teachers and trainers (SD = 0.60) [31]. With an estimated error (d) of 0.14 for gender stereotypes, the required sample size for a confidence interval of 99% was 127 subjects.

The selection of the participants was made through a consecutive non-probabilistic sampling, selecting all possible subjects who were accessed who wanted to participate voluntarily and who met the following inclusion criteria: (1) acting as a trainer or teacher of adolescents at the time of the investigation; (2) completing the questionnaire “beliefs and gender stereotypes towards physical activity and sport (CEGAFD)” [32]; (3) providing a completed informed consent form. The final participants included 49 teachers (35 males and 14 females; mean age: 36.27 ± 10.25 years old) and 78 trainers (53 males and 25 females; mean age: 26.71 ± 5.06 years old).

### 2.3. Procedure

To carry out the data collection, teachers and trainers completed the questionnaire “beliefs and gender stereotypes towards physical activity and sport (CEGAFD)” [32] anonymously and individually. Prior to this, they were informed about the importance of truthfulness in their answers. The participation of the teachers and trainers was completely voluntary. This is a semi-structured questionnaire that presents a Cronbach’s alpha of 0.899, as well as a confirmatory factor analysis with very satisfactory results (χ^2^/df = 4.47; RMSEA = 0.059, CFI = 0.95; GFI = 0.92; RMR = 0.064), which gives it acceptable reliability and validity. This questionnaire is composed of 24 items that allow information to be obtained on the sports lifestyle habits and gender stereotypes of the participants in relation to different areas of the sports and educational environment, classifying the results obtained in 5 dimensions: differences associated with gender and its relationship with physical activity; sport and gender; stereotypes about physical activity associated with gender; beliefs about physical activity and gender; physical education classes and gender. The first dimension refers to differences in the interests and possibilities of participation in physical activities between boys and girls; the second dimension indicates differences in the barriers and difficulties encountered by men and women in sport; the third dimension presents gender stereotypes commonly related to the sports field; the fourth dimension includes statements about the differences present in the possibilities of physical and technical development of boys and girls; and the fifth dimension refers to differences in the participation of boys and girls in physical education classes. A four-point Likert scale was used to provide answers according to intensity (1 = strongly disagree; 2 = disagree; 3 = agree; 4 = strongly agree). To determine the value corresponding to each factor, the value contributed, from 1 to 4, to each question of that dimension was summed following the methodology of Vera et al. [32]. The maximum score in the category “differences associated with gender and its relationship with physical activity” was 28 points; in “stereotypes about physical activity associated with gender”, it was 20 points, and in “sport and gender”, “beliefs about physical activity and gender”, and “physical education classes and gender” it was 16 points [32].

### 2.4. Data Analysis

The distribution of the data was initially assessed using the Kolmogorov–Smirnov normality test. Due to the normality of the data, statistical analysis was performed using parametric tests. To obtain the results, the Student’s t-test was performed to determine the differences in gender stereotypes between teachers and trainers and between men and women. Cohen’s d was calculated to determine the size of the effect in these cases, being small when d < 0.2, moderate when d < 0.8, and large when d > 0.8 [33]. Subsequently, a one-way analysis of variance (ANOVA) of one factor was carried out to determine the existence of differences in gender stereotypes between male and female teachers and male and female trainers, as well as a multivariate analysis of variance with covariates (MANCOVA) to establish these differences as a function of age and years of experience. Bonferroni’s pairwise comparison was performed setting the statistical significance value at *p* < 0.0125. The confidence interval (CI) of the differences (95% CI) was included. Partial eta squared (η^2^) was used to calculate the effect size (ES) and was defined as small if ES ≥ 0.10, moderate if ES ≥ 0.30, large if ES ≥1.2, very large if ES ≥ 2.0 [34]. A value of *p* < 0.05 was established to determine statistical significance. Statistical analysis was performed using the SPSS statistical package (v.25.0; SPSS Inc., Armonk, NY, USA).

## 3. Results

Table 1 and Table 2 present the results of Student’s t-tests to determine the differences in each dimension of gender stereotypes according to the profession and sex of the participants. Regarding the profession, the differences were significant in the dimension “beliefs about physical activity and gender” (*p* = 0.048), with teachers having the highest values, and in the dimension “physical education classes and gender” (*p* = 0.006), in which trainers reported the highest scores, with a moderate effect size in both cases (Table 1). Sex, on the other hand, showed differences in the dimensions “sport and gender” (*p* = 0.005), with females reporting higher scores, and “beliefs about physical activity and gender” (*p* = 0.045), with men scoring higher in this case and with a moderate effect size in both cases (Table 1). Table 2 shows the results of the ANOVA and MANCOVA. Regarding the ANOVA results, the differences between male teachers, female teachers, male trainers, and female trainers were significant in the dimensions “sport and gender” (*p* = 0.023), “beliefs about physical activity and gender” (*p* = 0.015), and “physical education classes and gender” (*p* = 0.039), with a moderate effect size in all three dimensions. Female teachers showed the highest values in “sport and gender”, male teachers in “beliefs about physical activity and gender”, and female trainers in “physical education classes and gender”. When considering the covariates age and years of experience in the MANCOVA, age showed significant differences in “sport and gender” (*p* = 0.029), while years of experience showed differences in “beliefs about sport and gender” (*p* = 0.044), with a moderate effect size.

Bonferroni post hoc analysis of the ANOVA and MANCOVA results was carried out. The differences were significant between male and female teachers in the dimensions “sport and gender” (*p* = 0.008; mean diff = −2.186; 95% CI = −3.790; −0.581), with female teachers scoring higher, and “beliefs about physical activity and gender” (*p* = 0.012; mean diff = 1.329; 95% CI = 0.295; 2.362), with stereotypes of male teachers being higher, and between male trainers and male teachers in “beliefs about physical activity and gender” (*p* = 0.012; mean diff = −0.920; 95% CI = −1.632; −0.208), with the highest score for male teachers, and in “physical education classes and gender” (*p* = 0.024; mean diff = 1.475; 95% CI = 0.200; 2.751), with male trainers scoring higher. When the covariates were included in the analysis (MANCOVA), age showed differences in “sport and gender” between female trainers and female teachers (*p* = 0.043; mean diff = −1.832; 95% CI = −3.608; -0.056), and between male teachers and female teachers (*p* = 0.010; mean diff = −2.087; 95% CI = −3.669; −0.504), with female teachers score higher in both cases. Regarding years of experience, the differences were significant in “beliefs about physical activity and gender” between male teachers and female teachers (*p* = 0.013; mean diff = 1.329; 95% CI = 0.291; 2.367) and male trainers and male teachers (*p* = 0.015; mean diff = −0.935; 95% CI = −1.682; −0.188), with male teachers scoring higher in both.

## 4. Discussion

The first aim of this research was to establish differences in gender stereotypes between teachers and trainers according to profession and sex. In terms of profession, the results obtained showed significant differences, with teachers showing more stereotypes in the dimension “beliefs about physical activity and gender” and trainers in the dimension “physical education classes and gender”, this finding being relevant because it shows that gender stereotypes are still present in teachers and trainers, which coincides with the results of previous research [13,19,21,22]. The dimension “physical education classes and gender”, in which trainers scored higher than teachers, refers to the fact that adolescent boys benefit to a greater extent than girls from physical education classes, mainly due to the way in which the activities are presented by the teachers and the order in which the players are chosen when forming teams [32]. The dimension “beliefs about physical activity and gender”, in which teachers obtained higher scores than trainers, addresses the differences in the possibilities of physical development of boys and girls. The importance of these results is due to the fact that trainers showed gender stereotypes related to the educational field, while teachers presented those corresponding to the general sports field, so that professionals in each area attributed blame to the rest of the areas, but not to their own, as had been indicated in previous research [13,18,20]. This could be due to the fact that trainers maintain a generalized view that physical education is a masculinized field in which activities that favor only the male sex are proposed [16,19]. Therefore, it is essential that teachers and trainers analyze the values, beliefs, and behaviors they transmit to young people attending schools and sports teams, becoming aware of the role they play in the transmission of gender stereotypes, since if the aim is to eradicate them completely from the sports environment, changes must begin to be made in these aspects of the hidden curriculum of professionals working with adolescents.

The trainers, therefore, have a vision in which the gender stereotypes of adolescents are promoted by the educational environment, but it is surprising that in teachers the dimension with the highest score is “beliefs about physical activity and gender”, in which the possibilities of physical development of adolescents are valued, without mentioning any specific area. The subject of physical education reports different levels of sports practice between boys and girls, with boys showing higher levels of moderate and vigorous physical activity [35]. These levels of practice vary according to the content of the classes [35] and, despite the diversity of activities proposed according to official plans and programs, collective sports characterized by competitiveness occupy a large part of the teaching planning of educational centers, encouraging the practice of boys and decreasing that of girls [19]. In contrast, sports teams are mostly composed of adolescents who are motivated to practice sports on a regular basis [36,37], so trainers are more likely to observe physical and sporting development in boys and girls, avoiding the presence of gender stereotypes in this area, which is consistent with the results found in the research. This finding was unexpected and relevant due to the fact that extracurricular sport is voluntary, so the presence of stereotypes could be more frequent in certain modalities in which boys and girls still have a stereotypical perception, but physical education is not voluntary and participation and physical development should be similar in all students.

Regarding the sex of the participants, it should be noted that the results showed differences between men and women in the dimensions “sport and gender” and “beliefs about physical activity and gender”. Women, mainly female teachers, compared to male teachers, showed higher scores in the dimension “sport and gender”, which refers to the difficulties encountered by girls in practicing sports compared to boys. These data should be interpreted with caution, but a possible explanation would be that female teachers had an adolescence in which female sport was unusual and received numerous sexist criticisms [38,39], being common that female sport practice was limited. This perception regarding female sports practice has been maintained for decades, as observed in the results of the present research, and if it were corroborated in future research that female teachers present gender stereotypes related to the difficulties that girls have in practicing sports, it would be a fundamental element to take into account in teacher education programs, since the perception by female teachers that women have more barriers to sports practice could be transmitted to the adolescents they teach [37,40], thus maintaining gender stereotypes in this area. Likewise, future research would be necessary to address the differences in the barriers perceived by adolescent girls who only attend physical education classes and by those who, in addition, practice an extracurricular sport, in order to clarify the real influence of female teachers.

Regarding the dimension “beliefs about physical activity and gender”, male teachers reported higher values compared to female teachers, which is especially relevant because it corroborates the finding that in the educational environment there are still stereotypes that come from the male gender and are related to the ability of adolescent boys and girls to develop physically. The contents of physical education classes are a fundamental element that influences the levels of participation of adolescent boys and girls [35], to which the possibility must be added that teachers, through their behavior, or the ways of organizing the class in which boys and girls practice separately, may transmit to their female students a misconception of the existence of differences between the sexes. However, the gender of the teacher is also relevant, since previous research has observed that adolescent boys and girls attach greater importance to physical activity when they have a female teacher, compared to when they have a male teacher [41,42]. This situation could favor the increased practice of girls during physical education classes led by a female teacher, compared to when they are led by a male teacher, and this could be the reason why teachers perceive greater possibilities for the physical development of boys compared to girls.

The second aim of the present research was to analyze the influence of age and years of experience on the gender stereotypes of male and female trainers and male and female teachers. The results showed significant differences between male and female teachers and between female trainers and teachers in the “sport and gender” dimension when considering age, with female teachers reporting higher values in both cases. Regarding the years of experience in the work environment, the differences were significant in the dimension “beliefs about physical activity and gender” between male and female teachers and between male trainers and teachers, with male teachers scoring higher in both cases. These results are similar to those found in previous research in which age was a relevant factor for the emergence of gender stereotypes, with older adults showing higher gender stereotypes compared to younger adults [43]. A possible explanation for these results would be that adults live daily in work and personal environments that are characterized by the presence of certain gender stereotypes [44,45], to which the influence exerted by other elements present in everyday life such as the language used [46] or the information provided by the media must be added [47,48], progressively increasing adults’ perception of gender stereotypes present in all areas, including sport. Another possible explanation would be that male and female teachers with older age and work experience are those who have lived during their childhood and adolescence in a society in which stereotypes were present in all social spheres but were not perceived because they were considered normal at that time. In today’s society in which younger male and female teachers have developed and been educated, there have been social changes of great relevance that have allowed progress in the field of gender stereotypes, being these new generations those who have a clearer vision of how to achieve the equality that past generations did not have, thus favoring an egalitarian physical education in which boys and girls develop in a similar way.

With regard to the main practical implications of the results obtained, it should be noted that teachers and coaches present gender stereotypes related to sports practice, so that it would be necessary for the training courses given to teachers and sports coaches to make professionals aware of the influence they can exert, even unconsciously, on the adolescents with whom they work. The results obtained in the research allow us to identify more precisely the areas in which each professional presents more stereotypes, serving as a starting point to provide information to these professionals about the beliefs and thoughts that can generate greater gender stereotypes in adolescents within their field of work, favoring a more inclusive practice without discrimination in the educational and sports environment, regardless of sex, skill level, or performance of the adolescents. Furthermore, the differences in participation in research between men and women is a noteworthy aspect that should be taken into account by the institutions that promote training courses mainly related to the sports coaching profession, encouraging more girls to work as sports coaches, as this will allow more adolescent girls to practice sports and become interested in the profession of sports coaching.

Considering the main limitations of the study, it should be noted that the number of men and women was quite disparate within the sample, which is similar to what found in previous research, indicating that there are fewer female coaches than male coaches, probably due to the reduced number of previous mentors for girls, as well as to the difficulty in reconciling work and family life, generating a work environment with greater male participation [23,25,26]. This aspect should be taken into account in future research, if a similar sample is to be obtained for both sexes. Therefore, these results are a first approximation of the gender stereotypes that may be present in teachers and trainers working with adolescents; therefore, it would be interesting for future research to follow the same line, providing more scientific evidence to understand whether the role played by teachers and trainers is similar or whether, on the contrary, one of the groups is more relevant. To this end, it would also be interesting to consider the assessment that adolescents make of the stereotypical activities of their teachers and trainers, including the opinions of adolescents who only practice physical education and of those who also practice extracurricular sports.

## 5. Conclusions

To conclude, the results obtained confirm the presence of gender stereotypes in trainers and teachers of adolescents. Female trainers have the perception that physical education classes are still stereotyped; female teachers consider that the sports field has stereotypes that favor the practice of sports by boys and hinder that of girls, while male teachers think that there are differences in the possibilities of physical development of boys and girls. In addition, teachers and trainers who are older and have more years of experience have more gender stereotypes. Therefore, it seems evident that trainers and teachers present gender stereotypes, but they consider that the transmission of these stereotypes to adolescents takes place in other spheres far from their work performance.

## Figures and Tables

**Table 1:** Gender stereotypes present in trainers and teachers.

	Profession					Sex				
	Trainers (*n* = 78)	Teachers (*n* = 49)	t	*p*	Effect Size (d)	Males (*n* = 88)	Females (*n* = 39)	t	*p*	Effect Size (d)
Differences associated with gender and its relationship with physical activity	12.12 ± 3.04	11.69 ± 3.23	0.743	0.459	0.14	12.20 ± 3.30	11.38 ± 2.56	1.377	0.171	0.28
Sport and gender	11.27 ± 2.42	11.51 ± 2.97	−0.501	0.618	0.09	10.93 ± 2.66	12.33 ± 2.31	−2.844	0.005	0.56
Stereotypes about physical activity associated with gender	9.87 ± 2.57	10.33 ± 3.41	−0.854	0.394	0.15	10.24 ± 3.10	9.62 ± 2.44	1.112	0.268	0.22
Beliefs about physical activity and gender	10.55 ± 1.63	11.16 ± 1.76	−1.995	0.048	0.36	10.99 ± 1.87	10.33 ± 1.16	2.025	0.045	0.43
PE classes and gender	8.24 ± 3.03	6.73 ± 2.81	2.811	0.006	0.52	7.49 ± 3.01	8.05 ± 3.06	−0.967	0.335	0.18

**Table 2:** Differences in gender stereotypes present in male trainers, female trainers, male teachers, and female teachers.

	Sex × Profession							Sex × Profession × Age			Sex × Profession × Years of Experience		
	Male Trainers (*n* = 53)	Female Trainers (*n* = 25)	Male Teachers (*n* = 35)	Female Teachers (14)	F	*p*	Effect Size (η^2^)	F	*p*	Effect Size (η^2^)	F	*p*	Effect Size (η^2^)
Differences associated with gender and its relationship with physical activity	12.45 ± 3.01	11.40 ± 3.03	11.83 ± 3.71	11.36 ± 1.50	0.910	0.438	0.22	1.193	0.277	0.31	0.050	0.823	0.25
Sport and gender	10.96 ± 2.49	11.92 ± 2.16	10.89 ± 2.95	13.07 ± 2.46	3.303	0.023	0.75	4.886	0.029	1.10	0.003	0.955	0.94
Stereotypes about physical activity associated with gender	10.21 ± 2.70	9.16 ± 2.15	10.29 ± 3.67	10.43 ± 2.77	0.982	0.404	0.23	2.013	0.159	0.39	3.483	0.064	0.24
Beliefs about physical activity and gender	10.62 ± 1.79	10.40 ± 1.26	11.54 ± 1.87	10.21 ± 0.98	3.637	0.015	0.81	2.438	0.121	0.99	4.162	0.044	0.82
PE classes and gender	8.08 ± 3.16	8.60 ± 2.75	6.60 ± 2.57	7.07 ± 3.43	2.871	0.039	0.65	0.007	0.933	0.65	0.121	0.728	0.66

## Data Availability

The data sets generated during and/or analyzed during the current study are available from the corresponding author on reasonable request.
